# Aetiology and risk factors for head and neck cancer: United Kingdom National Multidisciplinary Guidelines

**DOI:** 10.1017/S0022215116000360

**Published:** 2016-05

**Authors:** R Shaw, N Beasley

**Affiliations:** 1 Department of Molecular and Clinical Cancer Medicine, Institute of Translational Medicine, University of Liverpool, Aintree University Hospitals NHS Foundation Trust, Liverpool, UK; 2 Department of Otolaryngology – Head and Neck Surgery, Nottingham University Hospitals NHS Trust, Nottingham, UK

## Abstract

This is the official guideline endorsed by the specialty associations involved in the care of head and neck cancer patients in the UK. It discusses the aetiology and risk factors for head and neck cancer and the recommended interventions appropriate for each risk factor.

**Recommendations:**

• Recent evidence synthesis from National Institute for Health and Care Excellence suggests that the following brief interventions for smoking cessation work should be used:
  ○ Ask smokers how interested they are in quitting (R)
  ○ If they want to stop, refer them to an intensive support service such as National Health Service Stop Smoking Services (R)
  ○ If they are unwilling or unable to accept a referral, offer a stop smoking aid, e.g. pharmacotherapy. (R)
• Brief interventions are effective for hazardous and harmful drinking. (R)
• Specialist interventions are effective in people with alcohol dependence. (R)
• Most people with alcohol dependence can undergo medically assisted withdrawal safely at home, after risk assessment. (R)
• Management of leukoplakia is not informed by high-level evidence but consensus supports targeted use of biopsy and histopathological assessment. (R)
• The management of biopsy proven dysplastic lesions favours:
  ○ advice to reduce known environmental carcinogens such as tobacco and alcohol (R)
  ○ surgical excision when the size of the lesions and the patient's function allows (R)
  ○ long-term surveillance. (R)
• Fanconi anaemia patients should:
  ○ be followed up in a multidisciplinary specialist Fanconi anaemia clinic (G)
  ○ have quarterly screening for head and neck squamous cell carcinoma and an aggressive biopsy policy (G)
  ○ receive prophylactic vaccination against high risk human papilloma virus (G)
  ○ receive treatment for head and neck squamous cell carcinoma with surgery alone where possible. (G)

## Introduction

The major risk factors for head and neck cancer in the UK are tobacco smoking and alcohol consumption and withdrawal of these environmental carcinogens remains the focus for primary and secondary prevention. Additionally the role of human papilloma virus (HPV) is being increasingly recognised, but as the natural history and transmission of oral and oropharyngeal HPV infection are incompletely understood, the opportunities for reducing this risk are not yet clear. Some patients have recognised local or systemic pre-malignant conditions which are also discussed.

## Smoking

Smoking is an independent risk factor for head and neck cancer.^1^ Patients who continue to smoke during radiotherapy are more likely to develop osteoradionecrosis and to require hospitalisation during treatment. Continued smoking through radiotherapy was thought to have an adverse effect on local control (hazard ratio 1.5) and survival (hazard ratio 1.7), but more recent evidence would suggest baseline smoking status is more important.^2^ Smoking cessation before surgery is desirable to reduce the risk of anaesthetic related complications and improve wound healing, particularly after reconstructive surgery.^3^^,^^4^

Quitting tobacco smoking for a short period of time (one to four years) results in a head and neck cancer risk reduction of about 30 per cent compared with current smoking, reduces the risk of laryngeal cancer by 60 per cent after 10–15 years and after 20 years can reduce the risk of developing oral cavity cancer to the level of a never smoker.^5^

**Recommendations**

• Recent evidence from NICE suggests that the following brief interventions for smoking cessation work should be used:
  ○ Ask smokers how interested they are in quitting (R)
  ○ If they want to stop, refer them to an intensive support service such as NHS Stop Smoking Services (R)
  ○ If they are unwilling or unable to accept a referral, offer a stop smoking aid, e.g. pharmacotherapy (R)

## Alcohol

Alcohol is the other major independent risk factor for head and neck cancer. Patients who continue to drink heavily after treatment for head and neck cancer have a significantly worse quality of life^6^ and continued drinking has a negative impact on survival (hazard ratio 1.28).^7^^,^^8^ The beneficial effects of quitting alcohol, on the risk of developing head and neck cancer, are only observed after more than 20 years, when the level of risk reaches that of non-drinkers.^5^

Cessation of alcohol on admission for surgery can present a significant problem in heavy drinkers. A review in the British Medical Journal suggests that we should screen all patients for excessive alcohol consumption with a validated questionnaire such as Fast Alcohol Screening Test.^9^

**Recommendations**

• Brief interventions are effective for hazardous and harmful drinking (R)
• Specialist interventions are effective in people with alcohol dependence (R)
• Most people with alcohol dependence can undergo medically assisted withdrawal safely at home, after risk assessment (R)

## Human papilloma virus

Human papilloma virus -16 is an increasingly relevant causative agent in oropharyngeal and oral squamous cell carcinoma (SCC), however doubt remains in other sites and for other HPV subtypes. Combined data from recently published (2006–2009) studies shows that 55 per cent of 654 oropharyngeal SCC cases were HPV-16 positive.^10^ The prevalence of HPV-16 chronic infection in oropharyngeal mucosa of the general population is currently unclear. Without a clinically identifiable premalignant lesion, any future (primary or secondary) screening approach would rely on molecular biomarkers. Oral HPV infection increases with numbers of recent oral sex partners and isolated cases of transmission of HPV-16 between partners leading to the possible ‘transmission’ of cancer have been reported.^11^ Evidence seems currently insufficient to counsel avoidance of specific sexual activities, over and above guidance that informs the prevention of other sexually transmitted diseases. It is awaited with interest as to whether the current programme of vaccination against high risk HPV (strains 16 and 18) offered to 12–13-year-old girls will in the future reduce the incidence of head and neck squamous cell carcinoma (HNSCC).

## Premalignant lesions

Leukoplakia and erythroplakia are common premalignant lesions; however, most HNSCC cases have no history of such antecedent lesions. Biopsy-proven epithelial dysplasia is demonstrated in 25 per cent of biopsies of leukoplakia but most erythroplakia; however, HPV-16 is very rarely a factor in these conditions. The significant clinical predictors of malignant transformation in oral dysplastic lesions are non-smoking status, sub-site (e.g., high risk in lateral tongue and low risk in floor of mouth), non-homogeneous appearance, size of lesion greater than 200 mm and higher histological grade (severe *vs* mild/moderate). A recent systematic review of oral dysplasia (992 patients) showed malignant transformation in 12.1 per cent after mean 4.3 years following biopsy.^12^ Severity of dysplasia predicted for malignant transformation (*p* = 0.008). Lesions that were not excised demonstrated considerably higher transformation rate than those that were excised (*p* = 0.003).^13^ A binary histological grading into the high and low risks has been suggested based on good predictive power that has been independently verified in other series. A systematic review of laryngeal dysplastic lesions (942 patients) showed transformation in 14 per cent after a mean interval of 5.8 years, again severity of dysplasia correlated with risk of transformation.^14^

Importantly, these data only reflect patients already referred for a specialist opinion and with biopsy-proven dysplasia. In population-based studies of oral leukoplakia without histological inclusion criteria the risks are much lower; 40–50 per cent regress spontaneously and less than 1 per cent transform.^15^^,^^16^ There is insufficient evidence to justify screening in the general population to prevent oral cancer.^17^

The premalignant potential of oral lichen planus (OLP) is controversial; however, rigorously conducted retrospective series have confirmed the risk in classic inflammatory OLP with histological confirmation is low, at about 1 per cent. Oral lichenoid lesions which harbour features of OLP, but also epithelial dysplasia do present a modest risk for malignant transformation, and in some series this subset reflect the only cancer cases arising, interestingly with a predisposition to lateral tongue. Proliferative verrucous leukoplakia is a rare condition presenting with exophytic widespread progressive leukoplakia, somewhat refractory to intervention and with very high (50–80 per cent) transformation rates and hence, poor overall prognosis.

**Recommendations**

• Management of leukoplakia is not informed by high-level evidence, but consensus supports targeted use of biopsy and histopathological assessment (R)
• The management of biopsy proven dysplastic lesions favours:
  ○ advice to reduce known environmental carcinogens such as tobacco and alcohol (R)
  ○ surgical excision when the size of the lesions and the patient's function allows (R)
  ○ long-term surveillance (R)

## Premalignant conditions

### Inherited

Inherited conditions with increased risk of HNSCC include Fanconi anaemia (FA), ataxia telangiectasia, Bloom's syndrome and Li–Fraumeni syndrome. Fanconi anaemia has a very high risk of developing HNSCC (particularly oropharyngeal squamous cell carcinoma), most notably after haematopoietic stem cell transplantation.^18^ Recent evidence suggests a possibility that HPV may be implicated in FA-related oropharyngeal squamous cell carcinoma.^19^ Fanconi anaemia patients do not tolerate cisplatin and have severe toxicity with radiotherapy. Life expectancy has improved so that the population at risk for HNSCC is greater. Head and neck squamous cell carcinoma can occur early in patients as young as 11 years old. Further guidance is available from http://www.fanconianaemia.nhs.uk

**Recommendation**

• Fanconi anaemia patients should:
  ○ be followed up in a multidisciplinary specialist FA clinic (G)
  ○ have quarterly screening for HNSCC and an aggressive biopsy policy (G)
  ○ receive prophylactic vaccination against high risk HPV (G)
  ○ receive treatment for HNSCC with surgery alone where possible

### Acquired immunodeficiency

Patients who are immunosuppressed due to poor nutrition, advanced age, immunosuppressive therapy after transplant or acquired immunodeficiency syndrome (AIDS) are at greater risk of developing malignancy. The most commonly reported AIDS-related neoplasms of the head and neck region include Kaposi's sarcoma and non-Hodgkin's lymphoma. There is also an increased risk of oropharyngeal squamous cell carcinoma. Although HPV-related HNSCC has been seen in immunosuppressed patients, further clinical studies are needed to determine the safety and effectiveness of HPV vaccines in this setting.

**Key points**

• Smoking is an independent risk factor for head and neck cancer, is associated with post treatment complications and has an adverse effect on oncological outcomes
• Alcohol is an independent risk factor for head and neck cancer and continued drinking has a negative impact on survival
• High risk human papilloma viruses (HPV 16 and 18) are recognised causative agents for oropharyngeal squamous cell carcinoma
• Malignant transformation of oral dysplasia and laryngeal dysplasia occurs in 12 per cent (mean 4.3 years) and in 14 percent (mean 5.8 years) respectively.
